# Design and evaluation of a mobile application to assist the self-monitoring of the chronic kidney disease in developing countries

**DOI:** 10.1186/s12911-018-0587-9

**Published:** 2018-01-12

**Authors:** Alvaro Sobrinho, Leandro Dias da Silva, Angelo Perkusich, Maria Eliete Pinheiro, Paulo Cunha

**Affiliations:** 1 Federal Rural University of the Semiarid, Rodovia BR-226, Pau dos Ferros, 59900-000 Brazil; 2 Federal University of Alagoas, Av. Lourival Melo Mota, S/N Tabuleiro do Martins, Maceió, 57072-900 Brazil; 3 Federal University of Campina Grande, R. Aprígio Veloso, 882, Universitário, Paraíba, 58429-900 Brazil; 4 Federal Institute of Alagoas, R. Aprígio Veloso, 882, Universitário, Paraíba, 58429-900 Brazil

**Keywords:** Chronic kidney disease, Self-monitoring, Mobile application, mHealth, User-centered design

## Abstract

**Background:**

The chronic kidney disease (CKD) is a worldwide critical problem, especially in developing countries. CKD patients usually begin their treatment in advanced stages, which requires dialysis and kidney transplantation, and consequently, affects mortality rates. This issue is faced by a mobile health (mHealth) application (app) that aims to assist the early diagnosis and self-monitoring of the disease progression.

**Methods:**

A user-centered design (UCD) approach involving health professionals (nurse and nephrologists) and target users guided the development process of the app between 2012 and 2016. In-depth interviews and prototyping were conducted along with healthcare professionals throughout the requirements elicitation process. Elicited requirements were translated into a native mHealth app targeting the Android platform. Afterward, the Cohen’s Kappa coefficient statistics was applied to evaluate the agreement between the app and three nephrologists who analyzed test results collected from 60 medical records. Finally, eight users tested the app and were interviewed about usability and user perceptions.

**Results:**

A mHealth app was designed to assist the CKD early diagnosis and self-monitoring considering quality attributes such as safety, effectiveness, and usability. A global Kappa value of 0.7119 showed a substantial degree of agreement between the app and three nephrologists. Results of face-to-face interviews with target users indicated a good user satisfaction. However, the task of CKD self-monitoring proved difficult because most of the users did not fully understand the meaning of specific biomarkers (e.g., creatinine).

**Conclusion:**

The UCD approach provided mechanisms to develop the app based on the real needs of users. Even with no perfect Kappa degree of agreement, results are satisfactory because it aims to refer patients to nephrologists in early stages, where they may confirm the CKD diagnosis.

## Background

Early diagnosis of chronic diseases and monitoring of risk factors slow down the progression of diseases and may avoid adverse events (e.g., a sudden onset of renal disease in a person with hypertension) in the everyday life of patients. An undetected chronic disease results in several complications that put patients in injury-risk situations. The chronic kidney disease (CKD) and risk factors, such as diabetes mellitus (DM) and systemic arterial hypertension (SAH), are examples of diseases with high incidence [1]. The clinical situation of CKD patients not yet diagnosed is aggravating because it is usually asymptomatic. As a worldwide problem, CKD patients usually start their treatment in advanced stages where it is necessary dialysis and kidney transplantation, increasing morbidity and mortality rates, and healthcare costs [2, 3].

The CKD is defined as the reduction of the glomerular filtration rate (GFR) and the identification of proteinuria in urine [4]. Nephrologists use the GFR and the proteinuria to measure the level of the kidney function and to verify the excretion of protein/albumin in urine, respectively. They conduct analyses of these biomarkers through simple tests such as the estimation of the GFR using the Cockcroft-Gault [5] and modification of diet in renal disease (MDRD) [6] equations, and urine tests (e.g., dipstick test and albumin-to-creatinine ratio).

Simple tools are already available to assist physicians to measure kidney function and conduct risk stratification, such as the national kidney foundation (NKF) GFR web calculator [7] and the composite ranking for CKD risk evaluation of the kidney disease improving global outcomes (KDIGO) guideline [8]. These tools make the CKD diagnosis easier when physicians have access to them. However, this is not always the reality, mainly for developing countries. For example, Brazil, as a continental size country, has many issues related to computer-aided healthcare when compared to more developed countries that already use electronic health records (EHR), in special when considering primary care. Unfortunately, some remote locations even suffer from precarious public healthcare (sometimes with the lack of primary care physicians).

Additionally, some mobile applications (apps) for CKD diagnosis and monitoring can be found in the market. CKD-Go [9] is one example of an online app that assists users to verify their kidney function by inputting albumin-to-creatinine ratio and GFR values. Another example is the eGFR calculator app [10] (native for Android and iOS). It is possible to estimate the GFR using the Cockcroft-Gault, MDRD, CKD-EPI [11] and Schwartz [12] equations. Finally, the Kidney Disease Assistant [13] enables users to record test results and visualize their kidney function. Nevertheless, these apps have some limitations. For instance, the monitoring of CKD risk factors is not considered. On the other hand, well-accepted standards, such as the Health Level 7 (HL7) Clinical Document Architecture (CDA) [14], are not used to simplify the sharing of evaluation results during face-to-face consultations. This type of requirement is relevant considering that the CKD may be influenced by other clinical conditions and that evaluations usually need a second opinion.

The CKD diagnosis and monitoring are complicated tasks because nephrologists must analyze risk factors that influence the development and progression of the disease during risk evaluations. Chronic diseases of high incidence such as DM and SAH are relevant risk factors for the CKD development [15]. Abnormal values of glucose and blood pressure influence the treatment of the CKD negatively. CKD biomarkers of patients with DM and SAH should also be monitored to enable the diagnosis in early stages and avoid high morbidity and mortality rates.

When the CKD is diagnosed in less advanced stages, it is possible to reduce the need for dialysis and kidney transplantation, which consequently improves the quality of life of patients. A healthcare app may be used to monitor risk factors, record test results for CKD early diagnosis, and assist patients to follow the disease progression [16]. Considering a specific scenario, mobile health (mHealth) apps that generate personal health records (PHR) may be used to decrease problems related to primary healthcare at remote locations. Currently, mobile devices and Internet connectivity are spread worldwide and reach places that were inaccessible in the past. For instance, in Brazil, up to the present day, there are 280 million mobile devices with Internet connectivity (i.e., 1.4 devices per person) [17].

However, the development of this type of app has proven to be difficult due to the diversity of target users that range from young to elderly people. It is necessary to create apps to really fit users needs, and to be easy-of-use depending on the cognitive abilities of each group. A user-centered design (UCD) approach is an alternative to face these problems [18]. In this case, users needs and usability must be prioritized more than technological issues or choices.

Another important issue is that healthcare apps have a critical nature. Failures may induce physicians errors during diagnosis, monitoring, and treatment of patients. In this context, formal modeling languages are tools that can aid designers to improve the confidence on the specification of the system under development [19]. Coloured Petri nets (CPN) is an example of a formal language used to specify behaviors of complex systems [20]. Designers may carry out validation activities by means of techniques such as state space analysis and simulation, and verify safety properties. Once a non-ambiguous specification is generated, it may be followed by developers to decrease the likelihood of failures that may put patients in injury-risk situations. This issue is especially important because medical systems have to comply with strict regulatory requirements before commercialization. Formal models along with verification and validation results may be reused as safety and effectiveness evidence during a certification process [21]. The acceptance of model-based evidence has been increased by manufacturers and regulatory agencies such as the US food and drug administration (FDA) and the Brazilian national agency of sanitary vigilance.

Software systems have been designed to identify specific clinical conditions and assist physicians to diagnose and monitor chronic diseases taking into account relevant quality attributes [22–25]. For example, Pesl et al. (2016) [26] present the architecture and initial usability results of an insulin bolus calculator for diabetes. The system provides insulin recommendation, and it is composed of a smartphone app and a computer-based clinical platform. Petersen and Hempler (2017) [27] describe the development and test results of a mHealth app to assist diabetes self-monitoring. The app is tested by target users to evaluate usability and perceptions. In a CKD related work, Connelly et al. (2012) [28] describe the UCD of a dietary monitoring mHealth app for patients receiving hemodialysis. They carried out a pilot study using face-to-face interviews with 18 patients to evaluate the app. Perotte et al. (2015) [29] developed a risk prediction model to verify the CKD progression from stage 3 to stage 4. They conducted a statistical analysis to evaluate the model. However, despite the efforts and research advances in assisting diagnosed CKD patients, the identification of the disease in less advanced stages is still an ongoing issue that needs to be addressed. Additionally, other quality attributes (e.g., safety) should be considered during the design of mHealth apps.

In this article, a native mHealth app targeting the Android platform is presented as an approach to assist the early diagnosis and self-monitoring of the CKD. Risk assessments and monitoring are conducted based on internationally well-accepted medical best practices. The monitoring includes DM and SAH considering that they are two of the most relevant CKD risk factors. In addition, users are able to input and maintain information about prescribed medications, allergies, and test results. Information can be shared with physicians along with evaluation results during face-to-face consultations by means of CDA documents. One of the aims for adding the information management and self-monitoring of risk factors was to motivate the risk population to use the app and keep track of the CKD development and progression. A UCD guided the development lifecycle of the app that integrated traditional development process activities with the formal specification, formal validation, statistical analysis and usability test. This article contains research advances from [30, 31].

## Methods

The first activity of the UCD approach included literature reviews, interviews, and requirements validation to ensure that the system requirements reflect real needs of patients and physicians. The second activity includes an evaluation of the app considering effectiveness and usability. The study is composed of analyses of medical guidelines, in-depth interviews with health professionals, requirements specification, development and system evaluation.

### Medical guidelines

It is important to follow medical guidelines to ensure that internationally well-accepted best practices are incorporated in a mHealth app under development. Medical guidelines for the CKD diagnosis and the monitoring of DM and SAH in adults (*age*≥18) were analyzed in this research in the middle of 2012.

Two criteria were defined for selection of medical guidelines. The first criterion is related to the acceptance of the general community of nephrologists regarding the publisher of the guideline. The second one is the opinion of the experts interviewed during this research about the suitability of the guidelines in the context of developing countries. Specific requirements of each guideline were integrated to develop the app.

Therefore, three CKD medical guidelines were selected, including the KDIGO guideline [8], the national institute for health and care excellence guideline [32], and the kidney disease outcomes quality initiative (KDOQI) guideline [33]. Two medical guidelines were selected for DM and SAH, including the KDOQI clinical practice guideline for diabetes and CKD [34], the standards of medical care in diabetes [35], the KDIGO clinical practice guideline for the management of blood pressure in CKD [36], and the European society of hypertension (ESH) and European society of cardiology (ESC) guidelines for the management of arterial hypertension [37]. Physicians in developing countries usually follow the recommendations stated in well-accepted international guidelines.

### Interviews

Initial in-depth interviews with one nurse and four nephrologists were conducted between 2013 and 2014 in the nephrology center located at the university hospital of the Universidade Federal de Alagoas (UFAL), Brazil. The first development process activity of the app was also initiated in this period (i.e., the requirements discovery). Interviews guided this activity, initiated by the requirements elicitation from the selected medical guidelines. This was conducted to analyze and consider specific characteristics of developing countries that may be different from the more developed ones. Healthcare professionals were asked to keep contributing throughout all the remaining phases of the development process of the app.

### Specification

In the beginning of 2015, requirements elicited in the phase of initial interviews were formally specified with CPN to conduct more detailed analyses using the CPN/Tools software [30]. This type of specification is needed to reduce ambiguities, and it enables designers to verify safety properties of the app (when necessary) using state space analysis in state transition systems [38]. It is also possible to conduct requirements validation by means of model simulation to ensure that requirements reflect users needs. In this case, a nephrologist was trained to understand the formalism of CPN focusing on simulation capabilities of CPN/Tools. The simulation was important to identify issues in the specification before coding the app, aiming to decrease costs and development time.

### Development

The Java programming language was used to develop a native app focusing on the Android platform, in which personal and medical data are maintained using the SQLite database. The first version of the app was constructed and presented to the same nephrologist still in 2015. This aimed to make sure that all specified requirements were incorporated into the app. Afterward, a new version of the app was created according to the results obtained from the verification and validation activities outlined in the next sections.

### Evaluation

In addition to validating specific requirements, the app was evaluated as a complete system regarding statistical analysis and usability test. The evaluations were approved by the Brazilian ethics committee of the UFAL and carried out between 2015 and 2016.

#### Statistical analysis

Clinical data from the medical records of 60 patients composed the sample analyzed. Three nephrologists evaluated the medical records and provided their conclusions about the health situation of the subjects considering the risk of CKD development. A sample of the medical records from subjects with DM and SAH is presented in Table 1.

**Table 1:** Sample of medical records from subjects with diabetes and arterial hypertension

ID	SAH	DM	Creatinine	Urea	Microalbuminuria	Potassium	Weight	Age	Gender	GFR
1	X		0.86	35.8	36,8	4.4	71.5	56	F	96.9
2	X	X	0.54	25.2	0.6	4.1	80	60	F	139.9
3	X		0.81	33	77.5	3.9	61	79	M	63.8
4	X	X	0.6	33.4	5	4.2	87	49	F	155.7
5	X	X	2.06	65.1	26.9	4.4	101	74	M	36.6
6	X		2.3	41	28	4.4	67.4	50	M	36.6
⋮	⋮	⋮	⋮	⋮	⋮	⋮	⋮	⋮	⋮	⋮

Medical data were selected from records of 30 adult patients (*age*>=18) diagnosed with DM and/or SAH, and treated in the nephrology center located at the university hospital of the UFAL. Additionally, medical records of 30 adult patients not yet diagnosed with chronic diseases were selected to a control group. The sample analyzed included subjects with age between 31 and 79 years. In the first group, approximately, 94.5% were diagnosed with SAH where 58.82% of them were also diagnosed with DM.

During the data analysis, lack of data was identified due to the technological precariousness of the medical records located at the healthcare facility. The data was collected from the medical history recorded in a non-electronic format. Therefore, data related to proteinuria (more than 75% of the medical records) and other relevant data, such as weight, were not available. It required additional laboratory tests and other personal data (e.g., weight) to complete the medical records of the subjects. This reflects the issues faced regarding computer-aided healthcare along with an EHR infrastructure.

The Cohen’s Kappa statistic was used to analyze the evaluations conducted by three nephrologists and the app for the 60 medical records [39]. This statistical method was chosen because it can be used to measure the agreement between independent observers. The kappa calculation of the degree of subjects agreement is based on the difference between the expected and the observed agreement. The Cohen’s Kappa calculation is given by

$$ k = \frac{p_{o} - p_{e}}{1 - p_{e}} $$

where *p*_*o*_ is the observed agreement and *p*_*e*_ is the expected agreement. The kappa value is interpreted according to specific scales. A kappa value is classified as the absence of agreement when *k*<0; slight agreement when *k*≥0.01 and *k*≤0.20; fair agreement when *k*≥0.21 and *k*≤0.40; moderate agreement when *k*≥0.41 and *k*≤0.60; substantial agreement when *k*≥0.61 and *k*≤0.80; and almost perfect agreement when *k*≥0.81 and *k*≤0.99.

#### Usability test

A usability test was carried out by means of semi-structured interviews with each one of the 8 subjects randomly selected from the medical records analyzed. In total, 7 women and 1 man participated in the usability test, with the average age of 39 years. Six subjects completed high school as educational level, whereas 2 had not yet completed it. From the total subjects, 6 are young (age more than 18 and less than 35 years) and 2 elderly (age more or equal than 50 years) people.

A questionnaire was used to guide the semi-structured interviews. The first step of the test was an observational study where the subjects used the app freely and were asked to “think aloud”. In the second step, the target users were asked to answer some specific questions. The interviews were recorded electronically using computers along with text processors.

The applied questionnaire contained personal information and objective questions about ease-of-use, the usefulness of features, completeness, and clarity of information. For each objective question, some additional questions were asked to fully understanding and obtain justifications for each previous answer. Target users were asked to use the app in a controlled environment (a room of the healthcare environment). The primary objective questions provided for the users and a sample of the secondary ones are listed in Appendix.

## Results

In the development process of the app, the traditional process model was complemented to engage healthcare professionals and target users throughout all activities focusing on quality attributes such as safety, effectiveness, and usability. This was conducted following the five phases previously described. Outcomes obtained from these phases are outlined in the remainder of this section.

### System requirements elicitation

In the initial phase of the UCD, the primary work product generated, groups system requirements into a requirements document specification in natural language. Analyses of CKD medical guidelines (e.g., KDIGO) and the semi-structured interviews conducted, enabled the requirements discovery regarding the analysis of reference values of the primary CKD biomarkers named creatinine (between 0.6 and 1.4), urea (between 20.0 and 40.0), and potassium (between 3.5 and 5.5). These biomarkers aid physicians to identify abnormalities in the clinical situation of patients.

Proteinuria verification and GFR estimation showed to be commonly used methods to identify the CKD. High proteinuria levels indicate loss of renal function that can be verified classifying it as normal (less than 30), microalbuminuria (between 30 and 299) and albuminuria (higher than or equals to 300). When considering the GFR estimation, the Cockcroft-Gault equation was chosen instead of others such as the one proposed by the MDRD study because of the problem of identifying race in Latin American populations (a parameter used in the MDRD equation). This equation is also validated and widely used by physicians worldwide. The Cockcroft-Gault equation is given by

$$ C = \frac{(140 - I) * K}{P * 72} $$

where I is the age in years, K is the weight in kg, and P is the plasmatic creatinine. The result is calculated for men and women multiplying it by 1 and 0.85, respectively. Once the GFR is estimated, it is required the evaluation of the CKD stage and risk classification. These requirements were elicited according to the well-accepted KDIGO guideline classification (stages 1, 2, 3a, 3b, 4 and 5) and risk stratification (low risk, moderate risk, high risk and very high risk).

Afterward, reference values of blood glucose (preprandial, postprandial and fasting) and blood pressure (systolic and diastolic) were defined as system requirements to monitor DM and SAH (when a specific user has these clinical conditions). As described in the introduction of this article, DM and SAH are important risk factors that influence negatively the CKD development and progression.

Furthermore, given that physicians prescribe many medications for chronic patients, it is necessary to monitor and control the drug ingestion. The non-compliance with medications may complicate the clinical situation of patients during their treatment. It implied that the verification of allergic reactions to drug substances and also drug interactions composed the requirements specification of the app.

### Formal specification and validation

**Fig. 1:**
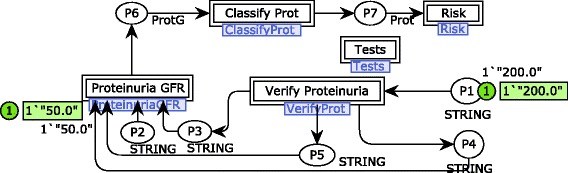
Main CPN module for the CKD risk identification. It contains a high level representation of the process defined to identify a possible risk for CKD

Given that medical systems are safety-critical, it is important to decrease ambiguities in the specification. Therefore, some of the requirements stated and initially represented with natural language were specified formally by means of the mathematical formalism CPN using the CPN/Tools software [40]. It included models for the GFR estimation, CKD risk identification, and analysis of blood glucose and pressure. However, just a sample of the formal models is presented due to space constraints. CPN models enable one to simulate system behaviors in order to validate requirements specified. Simulations were conducted along with a nephrologist to ensure that requirements really represent users needs in a modeling level. The main module for the CKD risk identification is shown in Fig. 1. Note that models are presented along with a sample of simulation to illustrate how the nephrologist validated the specification. In this case, the places named P1 and P2 were set with the initial markings 200.0 and 50.0 to represent proteinuria and GFR values.

**Fig. 2:**
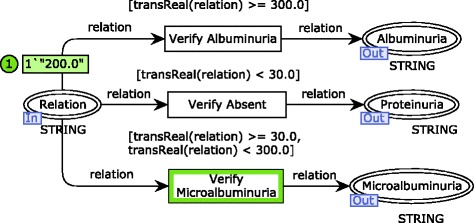
CPN module for the proteinuria verification. The first step of the risk identification process modeled

A first step is needed to verify if the proteinuria value relates to albuminuria and microalbuminuria (or none of them) in the substitution transition Verify Proteinuria. A module associated with this substitution transition is shown in Fig. 2. The proteinuria verification is conducted according to reference values indicated in the international medical CKD guideline analyzed [8]. For example, in this simulation sample, microalbuminuria is identified by the fire of the transition Verify Microalbuminuria that sends a token to an output port place connected to the substitution transition Proteinuria GFR.

**Fig. 3:**
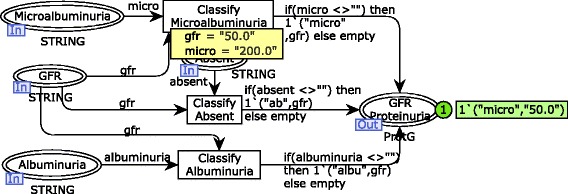
CPN module for the proteinuria and GFR relationship. The second step of the risk identification process modeled

Results of proteinuria verification are associated with GFR values in the substitution transition Proteinuria GFR to enable the CKD stage classification (see Fig. 3). Note that just one of the transitions Classify Microalbuminuria, Classify Absent and Classify Albuminuria will eventually fire, if and only if, a GFR value is defined and a proteinuria verification is available. This requirement is guaranteed because of the rule for transition firing that regards input places. It prescribes that there should exist tokens in each input place of such transition in a number more than the weight of input arcs. Considering the simulation sample, the transition Classify Microalbuminuria fires instead of its concurrent transition Classify Albuminuria due to the negative result obtained for albuminuria.

**Fig. 4:**
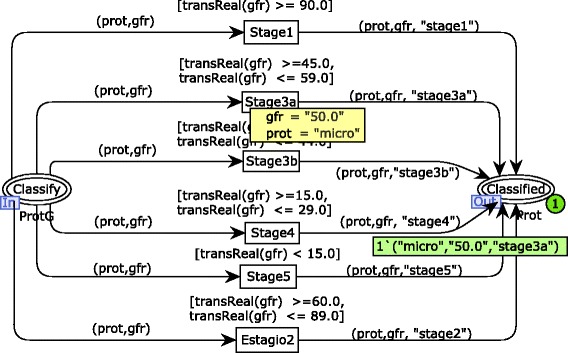
CPN module for the CKD stage classification. The third step of the risk identification process modeled

According to the requirements discovery, the CKD risk identification should be conducted based on the KDIGO classification. It relates the CKD stage classification and the verification of proteinuria. In order to simplify the specification, it was divided into two modules using the hierarchical feature of CPN. The module for the CKD stage classification is illustrated in Fig. 4 (substitution transition Classify ProtClassify Prot). The GFR value is verified to identify in which stage of the disease a subject is fitted. Once the GFR is related to the level of the disease progression, the final risk analysis is carried out. Note that, in the simulation sample, a subject is classified in the stage 3a considering that he/she has a GFR of 50.0. The output place (i.e., Classified) receives a composite token (tuple of 3 elements) that represents the product between proteinuria verification, GFR value and stage classification.

**Fig. 5:**
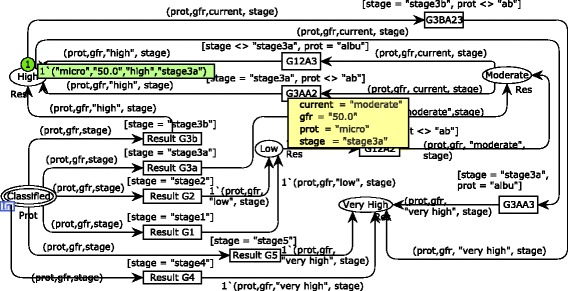
CPN module for risk analysis. The last step of the risk identification process modeled

The risk analysis specification also considers the risk classification of the KDIGO guideline (see Fig. 5). Therefore, there are four possible results for the risk analysis: low risk, moderate risk, high risk and very high risk. These possibilities are represented by state transitions triggered from the CKD stage classification model. One may see that, in this simulation sample, the subject being evaluated is classified at the stage 3a of the CKD with a high risk of the disease development. However, as stated above, the risk identification carried out in this approach is not limited to the model samples presented. Results obtained throughout the risk identification workflow modeled are combined to evaluate the current user’s situation, including DM, SAH, urea, creatinine, potassium, CKD stage and CKD risk analysis. The CKD risk identification process modeled was simulated covering a set of test cases related to the initial markings of the places P1 and P2 (see Fig. 1). Therefore, the hierarchical model was simulated at least 18 times to cover a minimum of 3 possible results (i.e., proteinuria, albuminuria, and microalbuminuria) associated with the 6 stages of the CKD (i.e., stages 1, 2, 3a, 3b, 4 and 5) along with the nephrologist.

Furthermore, the state space was calculated for each module of the CKD risk evaluation model to conduct a state space analysis using CPN/Tools. The standard reports of the CPN/Tools are composed of many statistics and properties related to the state space generated. However, only properties related to liveness, fairness, and boundedness were analyzed in order to improve confidence on the requirements specification. For example, a state space of 48 nodes was generated for the risk analysis module (see Fig. 5). In this case, there were no infinite occurrence sequences of transitions (fairness property). Moreover, transition instances never fired or always enabled were not identified (liveness property). Another important issue to be highlighted is that all dead markings found in the state space represent desired final states (i.e., 18 possibilities of CKD risk classification). Considering the boundedness property, it demonstrated that just one risk classification is obtained by means of the upper limit of 1 token for the places low, Moderate, High and Very High. It is possible to argue that model simulations enabled a more precise requirements specification, given that a nephrologist followed them and suggested improvements according to practical experience. Once the specification was completed, the state space analysis showed that the model reflected the improvements requested.

### Application

**Fig. 6:**
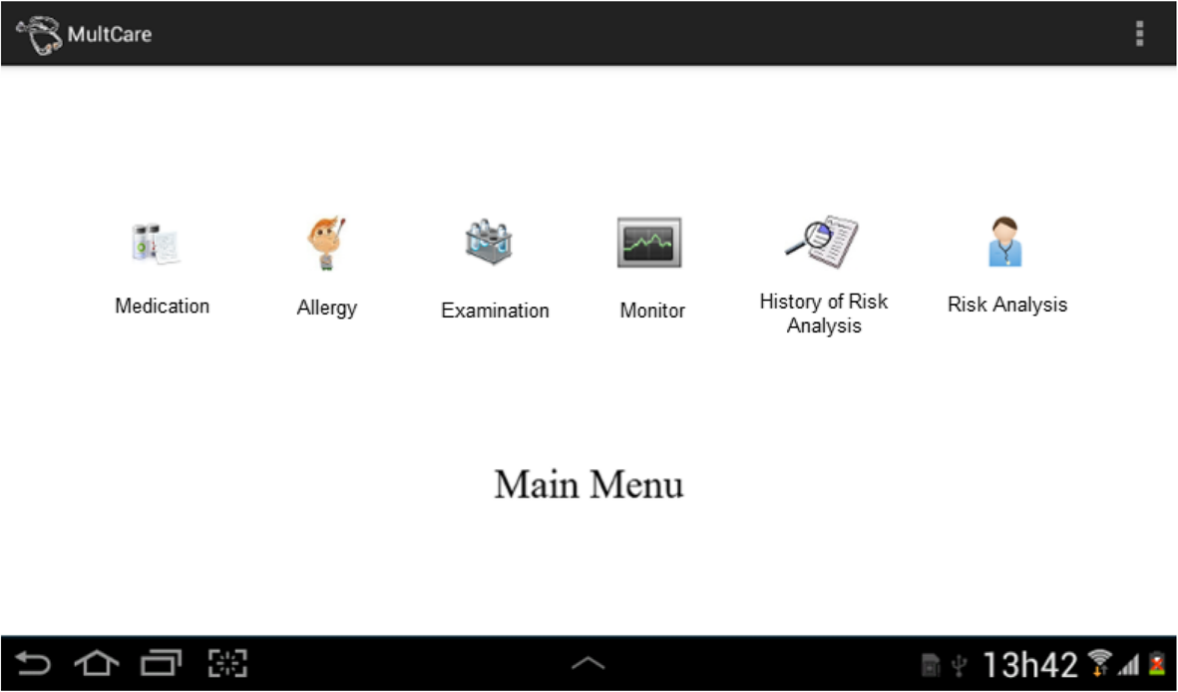
Main functionalities of the mHealth app. The app includes management of medication, allergy, and examinations. Additionally, self-monitoring capabilities and risk evaluations are available

The mHealth app was developed based on the formal requirements specification following the UCD approach defined. As outlined above, it aims to enable the self-monitoring of CKD risk by users outside a healthcare environment. The app periodically recommends patients to attend to a healthcare facility to conduct screening tests related to the CKD diagnosis. When users input all test results in the app, an analysis is conducted according to the formal requirements specification in order to identify a possible risk for CKD development. In a case of risk, the app is able to recommend the referral of patients to nephrologists in less advanced stages of the disease to avoid future complications in the clinical condition of patients due to late diagnosis. This is especially relevant when a patient resides in remote locations and is affected by precarious primary care (e.g., when medical specialists are not easily accessible). The main functionalities of the app are illustrated in Fig. 6.

It provides access control; management of medications, allergies, and examinations; monitoring of risk factors for CKD; history for CKD risk analyses; and CKD risk evaluation. Therefore, a PHR can be maintained by patients and provided to physicians in face-to-face consultations in order to improve evaluations. In the remaining of this section, the most important features are described. One may observe that the app is not limited to users not yet diagnosed with CKD. Patients already diagnosed and even physicians can be benefited using evaluation results to follow the disease progression.

**Fig. 7:**
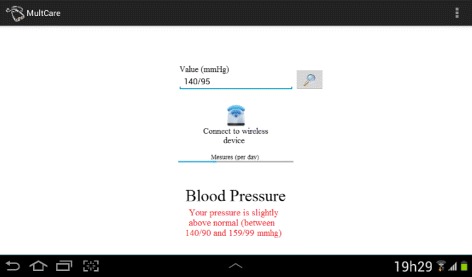
Arterial Hypertension Monitoring. Self-monitoring of hypertension manually or using a wireless mobile device

One of the important features discussed is the SAH monitoring. It is possible to verify the blood pressure manually or by means of a wireless sensor (see Fig. 7). In the first situation, the user reads results of the current blood pressure value from a device, fills out a text box and presses a button to ask for an evaluation. In the second case, the user activates the connection with a monitoring wireless device that automatically collects the current blood pressure value to present the evaluation. Evaluation results (presented at the bottom of the screen) are simple feedbacks about the current control of the disease.

**Fig. 8:**
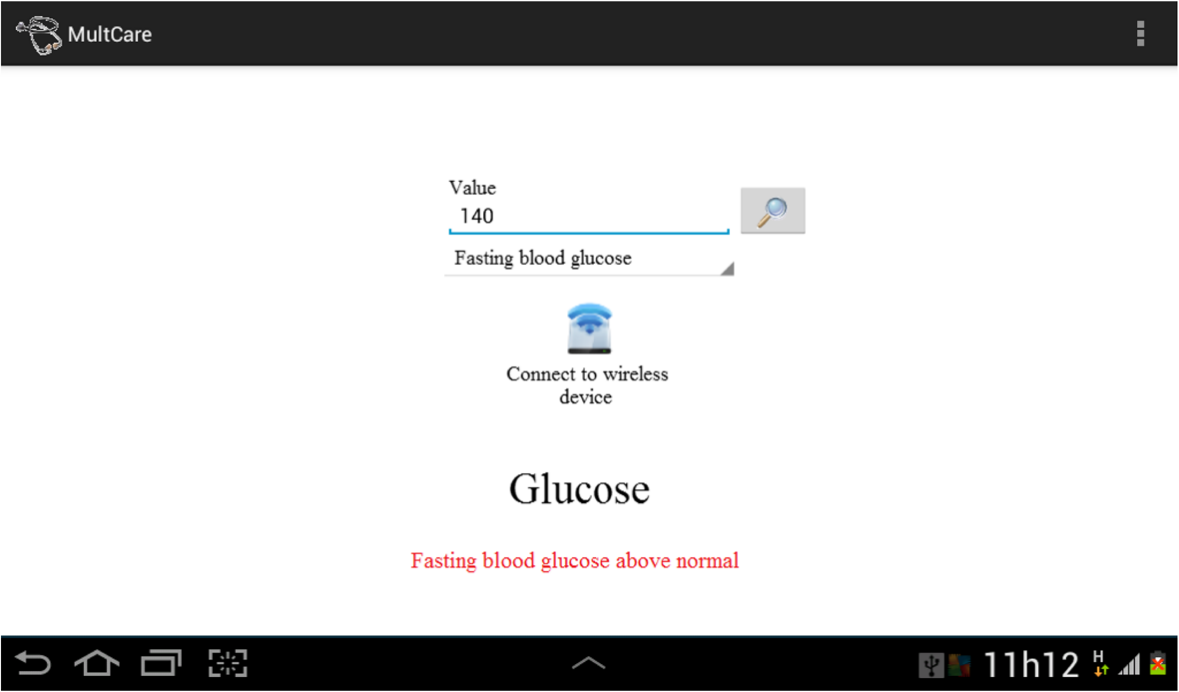
Diabetes Monitoring. Self-monitoring of hypertension manually or using a wireless mobile device. Preprandial, postprandial, and fasting glucose are verified

A similar approach is used to monitor DM. Users may verify their current situation, manually or not, by choosing it in the graphical user interface (GUI). However, in this case, it is considered that the user needs to verify blood glucose levels for preprandial, postprandial, and fasting glucose. Reference values for blood pressure and glucose are derived from international medical guidelines. The GUI for DM monitoring is illustrated in Fig. 8. Note that the result of the analysis is also shown at the bottom of the screen.

**Fig. 9:**
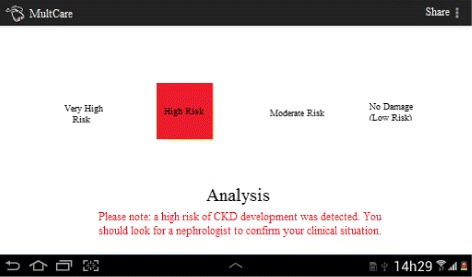
User interface for the risk evaluation. Four possible risk evaluation results are illustrated highlighting an example of high risk identified

On the other hand, when considering risk evaluations, results are divided into low risk, moderate risk, high risk and very high risk according to the formal requirements specification. The GUI of the app for the risk evaluation requirement is illustrated in Fig. 9. Note that an alert of a high risk of CKD is sent to the user in this sample of system usage. Moreover, it is possible to visualize a description of the risk evaluation by selecting the highlighted message (e.g., values of biomarkers and degree of the disease).

In addition, the app enables patients to share results with nephrologists when CKD risk evaluations are available. In this context, the well-accepted international standard HL7 CDA is implemented to generate clinical documents. A clinical document is a record that represents clinical and personal information of patients. This feature is important because evaluations can be exchanged with a specialist (or a set of specialists) to conduct the final diagnosis. The data sharing is carried out using a Bluetooth communication to facilitate the reuse of risk evaluations during face-to-face consultations.

### System validation

Formal requirements validation was an important part of the UCD approach because it prevented that misunderstandings regarding specific informal statements propagate throughout the development of the app. However, it is also important to analyze the system as a whole. In a first system validation scenario, a set of inputs were selected in order to conduct black box testing and analyze outputs using a statistical method. In a second validation scenario, subjects were selected randomly to participate in a usability test of the app to evaluate user satisfaction and perception.

#### Effectiveness evaluation

The statistical analysis was carried out by defining gross agreement, the Kappa concordance index and their respective confidence interval of 95% with no adjust of bias and prevalence. Data were analyzed considering two main aspects: CKD stage classification and renal damage risk. The three nephrologists classified the risk and stage of the CKD using the data collected from the medical records of the 60 patients. They analyzed the medical records considering data about risk factors (DM and/or SAH), urea, creatinine, potassium, microalbuminuria, weight, age, gender, and GFR. The same data was recorded in the app to obtain risk evaluation results. A sample of the CKD risk evaluation associated with the control group and conducted by the three nephrologists and the app is presented in Table 2.

**Table 2:** Sample of CKD risk evaluations for the control group

ID	Nephro 1	Nephro 2	Nephro 3	App
1	Low risk	Low risk	Low risk	Low risk
2	High risk	Moderate risk	High risk	Moderate risk
3	High risk	Moderate risk	High risk	Moderate risk
4	Low risk	Moderate risk	High risk	Low risk
5	Low risk	Low risk	Low risk	Low risk
6	Very high risk	Very high risk	High risk	Very high risk
⋮	⋮	⋮	⋮	⋮

Once the risk evaluation was provided by the three nephrologists and the app, the Cohen’s Kappa coefficient was applied to measure their agreement. The degree of concordance of the judges was analyzed separately for each one of the two main aspects: CKD stage classification and renal damage risk. The degree of concordance of the CKD stage classification showed a substantial concordance between the judges with a global Kappa *k*=0.7285 (column 2), with a significant *P*-Value (< 0.01). The Kappa results for the CKD stage classification are described in Table 3. Note that stage 4 presented *k*=1, representing a total agreement between the judges.

**Table 3:** Kappa results for CKD stage classification

Category	Kappa	Concordance Index (95.0%)	
Stage 1	0.6890	0.5440	0.8339
Stage 2	0.4795	0.2962	0.6626
Stage 3	0.9706	0.9099	1.0312
Stage 4	1.0000	1.0000	1.0000
Global Kappa	0.7285	0.5979	0.8589

Kappa values were also calculated for the renal damage risk. In this case, a substantial concordance was also obtained between the judges with a global Kappa *k*=0.7119 and statistical significance (*P*-Value < 0.01). The results for the renal damage risk are described in Table 4. Note that the major degree of agreement was obtained in the low risk category where *k*=0.8375 and an almost perfect agreement with *P*-value < 0.05. On the other hand, the moderate risk category presented the least Kappa value (0.2920). However, even with this issue, the global degree of agreement is still high.

**Table 4:** Kappa results of renal damage risk

Category	Kappa	Concordance Index (95.0%)	
Low risk	0.8375	0.7308	0.9442
Moderate risk	0.2920	0.0906	0.4930
High risk	0.7077	0.5780	0.8373
Very high risk	0.5234	0.0462	0.9984
Global Kappa	0.7119	0.5928	0.8308

#### Usability and perception evaluation

As stated above, in the second system validation scenario, the eight subjects were asked to use the features of the app freely at a room of the healthcare environment. A specific sample of test results randomly selected from the 60 medical records available was provided to patients in order to simulate a real-world situation and observe their behaviors when using the risk analysis capability.

As expected, it was possible to observe that there were differences between the young and the elderly people when using the app. Both groups presented different horizons of expectations and cognitive abilities regarding the app. Young subjects demonstrated greater capabilities to know and learn quickly about the use of the app without extensive training, stating that the app is easy-of-use (“It is easy to input test results!”). This scenario does not happen when the tests were conducted with elderly subjects. However, we do not consider it a critical issue because the main focus of the app is in screening risk of CKD development in early stages (i.e., young and middle-aged subjects).

Considering the self-monitoring capabilities, the answers were uniform and reviled that the monitoring of CKD biomarkers was the unique functionality not fully understood by the subjects (100% of negative answers). For instance, when asked to justify their answers about the creatinine biomarker, they argued that the use of this variable along with the GFR test is still not largely known in their everyday life (“We don’t know what the creatinine monitoring is!”).

When risk analyses were conducted, users were able to understand the risk alerts and the information regarding the advice to seek the second opinion of a physician (i.e., face-to-face consultation with a nephrologist). However, some technical information was not fully understood. Once details of evaluation results were accessed, it was identified that patients felt confused regarding the meaning of the CKD classification. More specifically, the stages 1, 2, 3a, 3b, 4, and 5, defined in the KDIGO guideline (“We don’t understand the meaning of these stages”).

## Discussion

Physicians who conduct the primary care of patients need more sophisticated and specific tools to aid them during patient evaluations. This is especially true in developing countries that suffer from lack of computer-aided healthcare. They usually conduct procedures needed to identify the CKD risk by hand or simple websites that do not provide enough information to assist a complete and precise diagnosis.

A mHealth app for risk evaluation and stratification of CKD can benefit both patients and physicians in managing and monitoring the disease, and in identifying a possible risk before critical health stages. In the case of CKD, even the number of screening tests being relatively small and of simple realization, they are enough for the initial identification of the CKD risk and the referral of patients to a nephrologist in early stages of the disease. Once this type of app is available, it may simplify the screening of the disease by providing the risk evaluation using internationally accepted medical guidelines.

Moreover, given that the app was developed following a well-accepted standard to represent and exchange clinical documents (i.e., the HL7 CDA), it is possible to reuse the initial CKD risk evaluation in further medical evaluations conducted by primary care physicians and/or nephrologists. Patients may share the app evaluation results by means of an initial CDA document and improve it in further clinical analyses. The UCD approach facilitated the decision of using this type of technology during the development of the app because it focused on real users needs and expectations.

However, there are some limitations to this study. For instance, some patients may be discouraged to use the CDA sharing functionalities of the app due to confidentiality concerns. Information sharing is essential to improve face-to-face consultations in remote locations. More study is needed to overcome this challenge regarding users confidence and acceptance.

Additionally, when looking at the results of the risk evaluations in the quantitative study, it is possible to observe that there are some disagreements between nephrologists and the app. Nephrologists expressed that this occurs due to the fact that they consider each aspect related to subjects separately, what does not happen in the app (“We take into account attributes, such as weight, age, and gender, separately”). Depending on each case, other attributes are evaluated during face-to-face consultations (e.g., SAH, urinary tract obstruction, and reflux and/or urinary tract infection). This type of attribute is known as a risk factor for the CKD progression.

Even with no almost perfect concordance between the app and the three nephrologists consulted, the results presented by the app are considered satisfactory because it aims the referral of patients to nephrologists (not a complete diagnostic), where they will be fully re-evaluated by a physician. Therefore, once the risk of CKD development is identified and the patient is referred to a physician, the results presented by the app can be reused during a complete diagnostic and more precise evaluation.

Regarding the usability test, it was possible to identify some needed GUI adaptations. For example, the creatinine monitoring functionality was changed to make it clear for users. However, there are also limitations to this test. For instance, the eight subjects may be considered a small sample. Additionally, the use of well-established and validated metrics defined in a usability test standard (e.g., ISO 9241-11 [41]) would improve the results obtained.

The app is currently in a second version guided by simple GUI adaptations. However, the evaluation results showed that further adaptations and testing are needed before the deployment of the app. As a future research direction, it is recognized the need to create some learning mechanisms to guide the users when handling the app and to better understand the CKD and risk factors (mainly for the elderly population). In this case, it is necessary to conduct further usability test by means of an internationally well-accepted standard. It is also envisioned the application of an Artificial Intelligence (AI) technique to improve the CKD risk evaluation activity conducted.

## Conclusion

Patients who used the app during the usability test found that it satisfied their expectations. The UCD approach conducted was important to ensure that real users needs are incorporated in the mHealth app. However, some functionalities regarding the self-monitoring need improvements. Additionally, focusing on quality attributes (e.g., safety, effectiveness, and usability) was an essential part of the design process. For example, it is currently recognized that healthcare apps should pass through some type of regulation to avoid hazardous situations to patients. The requirements specification generated by means of a formal modeling language (e.g, CPN) during the system specification phase may be a mechanism to generate safety and effectiveness evidence throughout the certification process.

## Appendix

**Table 5:** Questionnaire

Primary objective questions:
- Does the app provide an ease-of-use graphical interface?
Yes() No()
- Are the app descriptions of examinations compatible with descriptions
of laboratorial test results?
Yes() No()
- Are the features of the app useful?
Yes() No()
- Are the features of the app necessary?
Yes() No()
- Are the results of blood pressure monitoring clearly presented?
Yes() No()
- Are the results of blood glucose monitoring clearly presented?
Yes() No()
- Are the results of creatinine monitoring clearly presented?
Yes() No()
- Are the results of risk analysis clearly presented?
Yes() No()
- Is the information about risk analysis results enough?
Yes() No()
Sample of secondary questions:
- Are the test results easy to input in the app?
- Do you know what the CKD biomarkers mean?
- Do you understand the risk evaluation results?
- Do you understand what the CKD classification mean?

## Abbreviations

AI: Artificial intelligence
CDA: Clinical document architecture
CKD: Chronic kidney disease
CPN: Coloured petri nets
DM: Diabetes mellitus
EHR: Electronic health records
ESC: European society of cardiology
ESH: European society of hypertension
FDA: Food and drug administration
GFR: Glomerular filtration rate
GUI: Graphical user interface
HL7: Health level 7
KDIGO: Kidney disease improving global outcomes
KDOQI: Kidney disease outcomes quality initiative
MDRD: Modification of diet in renal disease
NKF: National kidney foundation
PHR: Personal health records
SAH: Systemic arterial hypertension
UCD: User-centered design
UFAL: Universidade Federal de Alagoas
